# A Depth Evidence Score Fusion Algorithm for Chinese Medical Intelligence Question Answering System

**DOI:** 10.1155/2018/1205354

**Published:** 2018-07-10

**Authors:** Xiabing Zhou, Binglin Wu, Qinglei Zhou

**Affiliations:** ^1^School of Computer Science and Technology, Soochow University, Suzhou, China; ^2^College of Information Engineering, Zhengzhou University, Zhengzhou, China

## Abstract

Question answering (QA) system is becoming the focus of the research in medical health in terms of providing fleetly accurate answers to users. Numerous traditional QA systems are faced to simple factual questions and do not obtain accurate answers for complex questions. In order to realize the intelligent QA system for disease diagnosis and treatment in medical informationization, in this paper, we propose a depth evidence score fusion algorithm for Chinese Medical Intelligent Question Answering System, which can measure the text information in many algorithmic ways and ensure that the QA system outputs accurately the optimal candidate answer. At the semantic level, a new text semantic evidence score based on Word2vec is proposed, which can calculate the semantic similarity between texts. Experimental results on the medical text corpus show that the depth evidence score fusion algorithm has better performance in the evidence-scoring module of the intelligent QA system.

## 1. Introduction

With the fast-paced development of artificial intelligence and natural language processing, studies on intelligent question answering systems have become a hot research topic in an increasing number of application fields. Intelligent question answering (QA) system is an application area of computer science, which attempts to build software systems that can provide accurate, useful answers to questions posed by human users in the natural language [1].

The medical information has drawn a wide attention from governments and industries because it is very effective in providing services to patients and society. Thus, the medical field has always been an important development direction of information. In order to promote the development of network medical services and meet the requirement of health consultation for users, it is needful to accelerate the research of intelligent question answering systems for the medical field. The medical intelligence QA system is an intelligent service software which is grounded on the large-scale medical health data and can accurately extract medical-related information to solve the problem of medical health for subscribers.

**Figure 1:**
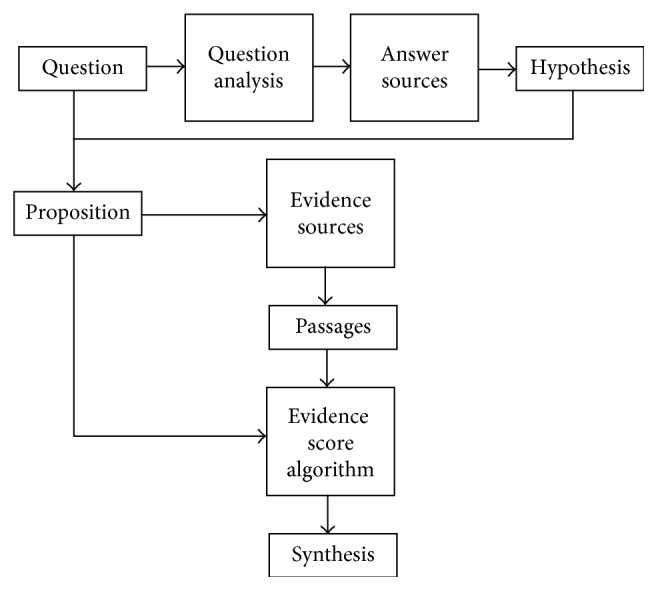
A brief flow of evidence score algorithm in the QA system.

The evidence score algorithm is a core algorithm of the whole intelligent QA system, and the algorithm is a measure that can quantify evidence related to each of the candidate hypothesis. The intelligent QA system can accurately output the optimal candidate answer through evidence scores of candidate answers. In the evidence search and scoring module of the intelligent QA system, the proposition is formed through both of questions input by the user and candidate answers retrieved by the system, and the *n*-paragraphs with high correlation are retrieved in the evidence sources. In the QA system, the location of the evidence score algorithm is shown in Figure 1.

It can be seen from Figure 1 that the evidence score algorithm is mainly located between the candidate answer generation module and the answer ranking module [2]. The scoring results, which can be obtained by calculating the similarity between the proposition and evidence passages in the evidence score algorithm, are used to measure the relevance of the hypothesis and question. The more accurate the candidate is, the more evidence it matches, and vice versa.

However, due to the diversity of Chinese semantics and both of the complexity and sensitivity of medical data, traditional single-evidence score algorithm cannot deal with Chinese medical data effectively in the Chinese medical intelligence QA system. In order to dig out the text information in depth and fully guarantee the quality of the evidence score, the evidence score of the proposition and the paragraph can be initially computed at each feature level. Each feature of the evidence score is accordingly combined into the text feature vector to import the depth neural network model which outputs the final evidence score results.

Based on the aforementioned method, this paper presents a deep evidence score fusion algorithm for Chinese medical intelligence QA system. Based on the ideas of the feature fusion and the feature selection [3], the depth evidence score algorithm integrates six kinds of evidence-scoring algorithms to calculate text feature scores of the proposition and the paragraph and provides the final evidence score through the depth neural network model. The text semantic evidence score algorithm in this paper is a new algorithm, which is based on Word2vec model. The new algorithm can compute the deep semantic similarity and deal with the matching of synonyms between propositions and evidence paragraphs. According to the experimental data and the analysis result, the depth text evidence score fusion algorithm has better scoring performance.

## 2. Related Work

Research on QA systems began as soon as the computer was born, as is well known in the Turing test. In recent years, the application of QA system in domain specific has greatly promoted its research and development, especially in the medical field. Three major QA approaches that focuse on the medical domain are deep Natural Language Processing (NLP) and Information Retrieval (IR) enhance shallow NLP and template-based QA [4]. At present, many scholars have been putting forward a large number of new QA systems based on these basic QA systems; for example, Athenikos et al. [5] have found a framework for a logic-based question answering system for the medical domain; besides, Lee et al. [6] have found the medical question answering system (MedQA) to better address the medical service. Compared to the English-oriented medical question answering system, the research of the Chinese-oriented medical question answering system started relatively late. The tools and methods currently applied to the Chinese-oriented QA system in the medical field are immature and are still in a preliminary stage of development. In many researches on Chinese-oriented medical question answering systems, it is possible to design novel tools and methods suitable for the Chinese environment, with drawing on the research results of the English-oriented medical question answering system and combining the actual conditions of the project. However, with the growth of information related to Chinese medicine and the increasing storage of data resources in the form of Resource Description Framework, question answering systems based on traditional retrieval techniques are gradually shifting to question-based systems based on semantic technologies. But, how to solve and improve the semantic accuracy of question answering system has always been a research challenge in this field, especially in the Chinese question answering system. The medical automatic question answering system based on the search engine primarily uses the traditional text similarity algorithm to calculate the matching score between texts. Nevertheless, the scoring results ignore the syntactic structure and semantic information of the text content. IBM DeepQA has good performance capabilities in dealing with massive heterogeneous medical data, especially at the level of data integration and reasoning. There are four evidence-scoring algorithms in the evidence-scoring module of IBM Watson Intelligent QA system, namely, Passage Term Match algorithm, Skip-Bigram algorithm, Text Alignment algorithm, and Logical Form scoring algorithm [7]. The proposition and paragraph of the structured data in the open domain can be scored in many ways; however, the IBM's evidence score algorithm has little effect on the evaluation of unstructured text data in Chinese medical field.

## 3. The Depth Evidence Score Fusion Algorithm

The text feature is an abstract method of abstracting the content of the text, which covers all the text information as much as possible [8]. In order to improve the accuracy of question answering system and consider the pros and cons of various QA systems synthetically, this paper proposes a deep evidence score fusion algorithm which is suitable for the evidence quiz module of intelligent QA system and carries out deeper text features between text comparison scores. In this paper, six text features including frequency, order, TF-IDF, syntactic, logical structure, and semantic can be mainly employed to evaluate the similarity degree of text information.

1. *The frequency feature of the text*: The word frequency feature refers to the frequency of the same entry between the matching proposition and the evidence paragraph.
2. *The order feature of the text*: The word order of the text is the matching score of the same entry sequence retrieved from texts.
3. *The TF-IDF feature of the text*: TF-IDF is the abbreviation of term Frequency-Inverse Document Frequency, which is one of the most important features of text processing in Natural Language Processing.
4. *The syntax feature of the text*: Syntactic analysis refers to the analysis of the grammatical functions of words in sentences, which is based on the syntactic dependency tree.
5. *The structure feature of the text*: According to the structural feature, Chinese sentences can be divided into single sentence and complex sentences. A complete sentence composition includes subject, predicate, object, attributive, adverbial, and complement. A sentence with the smallest unit as its entry, which is analyzed structurally, and then the corresponding sentence structure tree can be obtained. The similarity between sentence structures is calculated by introducing a convolution tree kernel function [9].
6. *The semantic feature of the text*: The semantic feature of the text can accurately reflect the meaning of texts or sentences through semantic analysis. Semantic analysis aims to transform the natural language understood by human beings into a formal language that can be understood by the computer. Hence, people communicate with the machine [10]. Semantic analysis can be divided into shallow semantic analysis and deep semantic analysis.

The depth text similarity fusion algorithm can mainly measure the text information from both of the proposition and evidence paragraphs at each feature level. Through filtering the text data, the text feature vector obtained by each evidence score algorithm is imported into the depth neural network model, and then the final evidence score is gained. The text features mainly include the six text features described earlier. Algorithms corresponding to each feature are text word frequency score algorithm, text word order score algorithm, text TF-IDF score algorithm, text syntax score algorithm, text structure score algorithm, and text semantic score algorithm. In order to reduce the noise interference and improve the weight of key entries [11], the preprocessing of word classification and word segmentation is added to each algorithm calculation.

In this paper, the text word order score algorithm is an improved algorithm based on the Waterman–Smith local DNA or amino acid sequence alignment algorithm [12]. Because the text score algorithm, text word order score algorithm, and text TF-IDF score algorithm are existing score algorithms based on the statistics, these algorithms are no longer described in detail in this paper. Text syntax score algorithm and the text structure scoring algorithm, which can dig out the similarity degree from the syntax features level and sentence structure level, are based on the syntactic analysis of dependency trees. Most of the calculation of text semantic similarity depends on HowNet [13] now. The text semantic evidence score algorithm, however, is based on the Word2vec study, which is a novel text semantic vector evidence score calculation method.

### 3.1. Text Semantic Evidence Score Algorithm Based on Word2vec

Deep learning has applications in many critical tasks in the QA system [14, 15], such as problem classification, answer selection [16, 17], and semantic matching. Word embedding is one of the neural network models in which the deep learning model is widely used in question answering systems and can be effectively used in the calculation of lexical semantics. The Word2vec Toolkit [18] utilizes the deep learning idea to provide an architecture that effectively implements the Skip-gram and CBOW models [19]. The model is trained efficiently on millions of levels of text data, and then all sentences are transformed into the *n*-dimensional semantic vector which can be used to represent the similarity of text semantic. The Word2vec toolkit also has a good application in the calculation of similar word, text clustering, and other aspects. Word2vec is trained based on the context of the sentences, and the spatial vector in the training model can contain the deep semantic information of the texts as much as possible.

The analysis of the text semantic evidence score algorithm based on Word2vec is described below.

In the large text corpus of medicine, through the Word2vec training model [20], each term in the length *s* text is mapped to the *l*-dimensional semantic vector and the vector dimension *l* = 300. Then the length *s* text is equivalent to *l* ∗ *s* semantic matrix, and the calculation of the semantic similarity is transformed into the operations of the vectorized matrix. Specifically, the proposition *Q* and the passage *P* are assumed. The entries set of the proposition *Q* are {*t*_1_, *t*_2_,…, *t*_*n*_}, and the entries set of the passage P are {*s*_1_, *s*_2_,… , *s*_m_}. In order to reflect the similarity between Q and P, the Q and the P are input into the Word2vec training model, the textual terms are transformed into l-dimensional semantic vector, so the proposition Q and the passage P are respectively transformed into semantic matrix *N* and semantic matrix *M*.

The matrix *N* = (*Vt*_1_, *Vt*_2_, …, *Vt*_*n*_), *Vt*_*i*_ = (*t*_*i*_ *l*_1_, *t*_*i*_ *l*_2_,…, *t*_*i*_ *l*_300_)^*T*^, *n* ≥ *i* ≥ 1; the matrix *M* = (*Vs*_1_, *Vs*_2_, … ,  *Vs*_*m*_), *Vs*_*j*_ = (*s*_*j*_ *l*_1_, *s*_*j*_ *l*_2_, …, *s*_*j*_ *l*_300_)^*T*^, *m* ≥ *j* ≥ 1. Calculating the cosine similarity between the *l*-dimensional semantic vector *Vt*_*i*_ and the *l*-dimensional semantic vector *Vs*_*j*_, the result of score is saved into the *l* ∗ *s* semantic matrix. Finally, the semantic matrix is as follows:

(1) $$\begin{array}{c} V_{1}={\left[\begin{array}{ccccc} Vt_{1}oVs_{1} & Vt_{1}oVs_{2} & \cdots & \cdots & Vt_{1}oVs_{m}\\ Vt_{2}oVs_{1} & Vt_{2}oVs_{2} & \cdots & \cdots & Vt_{2}oVs_{m}\\ \vdots & \vdots & \vdots & \vdots & \vdots \\ \vdots & \vdots & \vdots & \vdots & \vdots \\ Vt_{n}oVs_{1} & Vt_{n}oVs_{2} & \cdots & \cdots & Vt_{n}oVs_{m} \end{array}\right]}_{n\times m}, \end{array}$$

where *Vt*_*i*_ *oVs*_*j*_ represents the operation between the word vectors. That is,

(2) $$\begin{array}{c} Vt_{i}oVs_{j}=\frac{\sum_{a=1}^{300}t_{i}l_{a}\times s_{j}l_{a}}{\sqrt{\sum_{b=1}^{300}t_{i}l_{b}^{2}}\times \sqrt{\sum_{c=1}^{300}s_{j}l_{c}^{2}}}\;\left(n\geq i\geq 1,\;m\geq j\geq 1\right), \end{array}$$

where the *n* and the *m* represent the length of the text *Q* and the text *P*, respectively. From the sentence analysis of the text dependency tree, a serial number mark of each term is obtained in the current sentence. The serial number of textual terms and the corresponding semantic matrix *V*_1_ are combined, and the method of the most value of traversal matrix is as follows:

- The matrix *V*_1_ is traversed to find the global maximum value. If there are more than one maximum value, the corresponding serial number mark of matching terms are compared to select the smallest difference of serial numbers as the maximum value.
- The values of the ranks containing the maximum value return zero, then a new semantic matrix *V*_2_ is obtained.
- The semantic matrix *V*_2_ is repeated through the operation 1. Until the rank values of the semantic matrix *V*_min{*n*,*m*}_ are all zero, the semantic matrix *V*_1_ is iterated by min {*n*, *m*} times. The score formula of semantic similarity algorithm based on the Word2vec is recorded as follows:

(3) $$\begin{array}{c} \text{score}=\frac{\sum_{i=1}^{\min\left\{n,m\right\}}\max\left\{V_{i}\right\}}{\max\left\{n,m\right\}}. \end{array}$$

After the whole existing medical corpus is trained by the Word2vec model, each word in the matching texts is equal to a semantic vector that reflects the spatial mapping location of the word in the large corpus, so every word has a unique feature of vector representation. The smaller the space vector angle between the two terms is, the more similar the semantic level of the two terms is. Word2vec can also effectively match similar words or synonyms in the medical text. Through the calculation of the semantic similarity between the two terms, the semantic calculation between texts can be calculated in this way that we can regard the semantic of between the words as units. The result of the calculation is expanded in the form of a matrix. Through the selection of the maximum of rows and columns, the final score reflects the correlation between the semantics of the matching texts. A larger score means that two passages contain more semantic similarity terms in the specific medical text corpus. It can fully reflect the related degree between matching passages at a deep semantic level.

### 3.2. The Deep Neural Network Model

**Figure 2:**
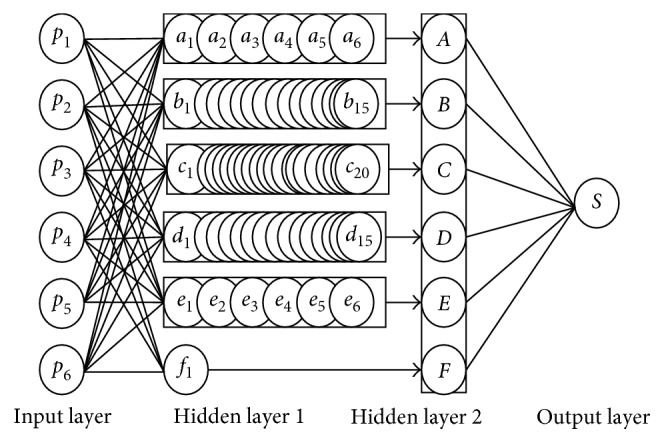
Depth neural network model.

The main feature of the deep neural network (DNN) [21] is an algorithmic model that can imitate the transmission between the human brain neurons and the pattern of the information processing. Because of the better nonlinear expression ability of the DNN, the DNN can fit a function between input features and output scores. The similarity information of two texts can effectively extract the similarity features of the textual information through each similarity degree algorithm. The similarity of the characteristics of each level between the matching texts are vectored as {*p*_1_, *p*_2_, *p*_3_, *p*_4_, *p*_5_, *p*_6_}, where *p*_1_, *p*_2_, *p*_3_, *p*_4_, *p*_5_, *p*_6_ represent the score results of text frequency evidence-scoring algorithm, text word evidence score algorithm, text TF-IDF evidence score algorithm, text syntactic evidence score algorithm, text structure evidence score algorithm, and text semantic evidence score algorithm, respectively. The depth neural network model is illustrated in Figure 2.

From Figure 2, a double hidden layer neural network model [22] is established, including an input layer, an output layer, and two hidden layers *H*_1_ and *H*_2_. Each layer is a feedforward neural network model [23], and the entire depth of the neural network can be regarded as the double hidden layer feedforward networks. The activation function is

(4) $$\begin{array}{c} \text{sig⁡}\mathrm{mod}\left(x\right)=\frac{1}{1+e^{-x}}. \end{array}$$

The characteristics of {*p*_1_, *p*_2_, *p*_3_, *p*_4_, *p*_5_, *p*_6_} are combined, and the number of combination of features is equal to *C*_6_^1^+*C*_6_^2^+*C*_6_^3^+*C*_6_^4^+*C*_6_^5^+*C*_6_^6^, so the hidden layer *H*_1_ has sixty-three hidden nodes which are {*a*_1_,…, *a*_*C*_6_^1^_}, {*b*_1_,…, *b*_*C*_6_^2^_}, {*c*_1_,…, *c*_*C*_6_^3^_}, {*d*_1_,…, *d*_*C*_6_^4^_}, {*e*_1_,…, *e*_*C*_6_^5^_}, and {*f*_*C*_6_^6^_}. A set of hidden nodes containing the same number of features is calculated by the corresponding neuron model, and six hidden nodes of the hidden layer *H*2 are obtained. The node value is introduced into the output layer neuron model, and then the final evidence score *S* is obtained.

The internal network is not fully connected; *v*, *w*, and *λ* represent the connection weights of the whole neural network. All implicit nodes for the implicit layer *H*1 and implicit layer *H*2 are calculated as follows:

Hidden layer 1:

(5) $$\begin{array}{c} \alpha_{s_{i}}=\overset{i}{\underset{j=1}{\sum }}v_{ij}p_{j},\;1\leq s_{i}\leq C_{6}^{i},\\ \alpha_{s_{i}}\in \left\{a_{s_{1}},b_{s_{2}},c_{s_{3}},d_{s_{4}},e_{s_{5}},f_{s_{6}}\right\}. \end{array}$$

Hidden layer 2:

(6) $$\begin{array}{c} \beta_{i}=\overset{C_{6}^{i}}{\underset{j=1}{\sum }}w_{ij}\alpha_{j},\;\beta_{i}\in \left\{A,B,C,D,E,F\right\}. \end{array}$$

The parameters *α* and *β* are case-sensitive correspondence (e.g., when *α*=*a*, *β*=*A*). The final score *S* can be obtained at the output layer, *S*=*λ*_1_ *A*+*λ*_2_ *B*+*λ*_3_ *C*+*λ*_4_ *D*+*λ*_5_ *E*+*λ*_6_ *F*. Then the total number of thresholds *θ* in the entire neural network is seventy. The deep network of greedy level training methods is used with supervised learning, and then the error back propagation algorithm (BP) is applied to reverse the entire network training [24].

In this paper, both of the training samples and the test samples are labeled as scoring data sets. The number of the training samples is 12000, and the number of test samples is 4000. According to the observation of similar degree between the texts, the score of textual similarity is artificially marked. It is easy to obtain the experimental data set that contains the scores of six features and a labeled score. The training is imported into the depth neural network to train the parameter weight of each layer in the neural network. At last, the fusion model of DNN is obtained. When the test set has verified, the accuracy of the fusion model of DNN can reach 78.49%.

## 4. Experimental Design and Analysis of Results

In this paper, the processing of word segmentation is completed by the ICTCLAS (Institute of Computing Technology, Chinese Lexical Analysis System) with JAVA version. Paragraph-search evidence is achieved by the Evidence Retrieval Technology [25], and the total number of the medical corpus text is initialized as *N*=18000.

The medical data are selected from the database of a Chinese medical website. For the further clarification of the algorithm, the medical proposition *Q* and the retrieved evidence paragraphs *P*1, *P*2, *P*3, and *P*4 are selected for the experiments of the depth of evidence score fusion algorithm, given an example as follows:

*Q*: “怀孕早期会有腹疼症状。” (Early pregnancy will have symptoms of abdominal pain.)
*P*1: “怀孕早期会有头晕乏力现象。” (Early pregnancy will have dizziness and fatigue phenomenon.)
*P*2: “慢性盆腔炎会有腹疼反应。” (Chronic pelvic inflammatory will have abdominal pain reaction.)
*P*3: “妊娠早期会有腹疼反应。” (Early pregnant will have abdominal pain reaction.)
*P*4: “怀孕早期有可能会有腹疼现象。” (Early pregnancy may have abdominal pain phenomenon.)

By processing and analyzing each document based on the word segmentation and the syntactic dependency tree, and completing the word preprocessing, the term set of proposition and passages can be obtained. Through the direct observation of the similarity of the textual features, the scoring result of each evidence is min {score (*Q*, *P*4), score (*Q*, *P*3)} > max {score (*Q*, *P*2), score (*Q*, *P*1)}. The score set {*p*_1_, *p*_2_, *p*_3_, *p*_4_, *p*_5_, *p*_6_} is obtained by calculating the evidence scoring of the proposition *Q* and paragraphs *P*1, *P*2, *P*3, and *P*4, and the score set is introduced into the trained deep neural network to get the final depth evidence scores *S*. According to the calculation based on the passage scoring algorithm, the experimental results are shown in Table 1.

According to Table 1, *S* (*Q*, *Pi*) represents that both proposition *Q* and passage *Pi* are calculated for each evidence score algorithm as algorithm import, which is *i* = {1, 2, 3, 4}. Based on the analysis of scoring results in Table 1 and the comparison of the values of the *p*_6_ between *P*2 and *P*3, it can be seen that the semantic evidence score algorithm of Word2vec has better performance on matching the “怀孕” (pregnancy) in text *Q* to the “妊娠”(pregnant) in text *P*3, than to the “慢性盆腔炎” (chronic pelvic inflammatory) in text *P*2. After the calculation of the depth evidence score fusion algorithm, the textual feature evidence score algorithm results and the final evidence score of the text *P*4 are higher than the corresponding scores of other texts; thus, the experimental test results and intuitive observations are consistent.

**Figure 3:**
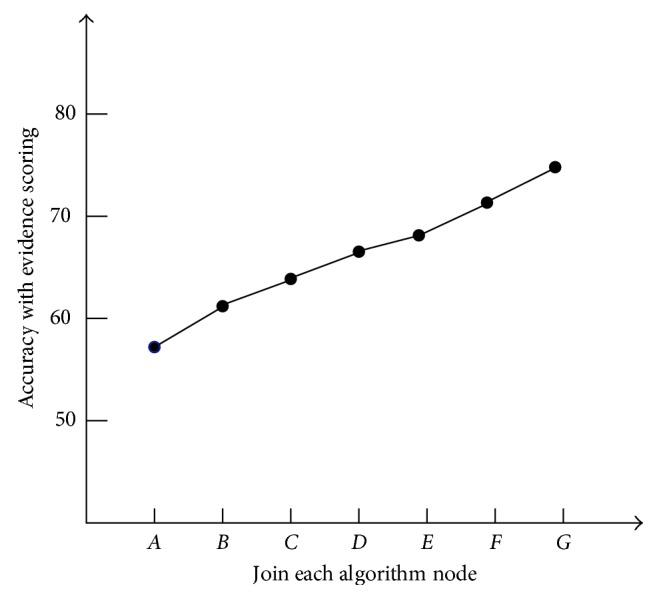
Accuracy with each evidence-scoring algorithm added.

To prove the validity and necessity of the evidence score algorithm in improving the performance of the QA system, a set of comparative tests was designed. There is a clear difference in the accuracy of the candidate answers, with or without the evidence score algorithm in a QA system. For the six evidence-scoring algorithms mentioned earlier, this paper adopts the method of successively adding each algorithm. The accuracy of the candidate answers given by the QA system under different conditions is taken as the criterion. As each evidence score algorithm is added, the change in accuracy is shown in Figure 3.

Point *A* represents the accuracy of the candidate answers given by the QA system without any evidence score algorithm. Starting from point *B*, based on the previous point, a new evidence score algorithm is added into the QA system each time. Given the six evidence-scoring algorithms described earlier, each algorithm is added to the evidence-scoring system in the order listed. The corresponding relationship between each node in the horizontal coordinate of Figure 3 and the added evidence evaluation algorithm is shown in Table 2.

According to Table 2, based on point *A*, text word frequency score algorithm is added at point *B*. Following this step, text word order score algorithm, text TF-IDF score algorithm, text syntax score algorithm, text structure score algorithm, and text semantic score algorithm are added at points *C*, *D*, *E*, *F*, and *G* in turn. Each evidence-scoring algorithm uses multiple threads to run concurrently. Before and after adding the algorithm system, the average time response of the system to answer questions increased from 1.25 seconds to 3.43 seconds.

From Figure 3, the accuracy of the system is gradually increasing as the evidence score algorithm continues to be added. When all six evidence score algorithms are added to the system, the accuracy is increased from 58.6% at point A to 73.7% at point G. In terms of above test results, it is very necessary to add the evidence score algorithm in the QA system.

In order to make a better illustration of the performance of the algorithm and the openness of the algorithmic system, the algorithm precision rate of some algorithm systems which are respectively Watson system evidence-scoring algorithm (WSESA) [26] and prominent feature extraction evidence gathering algorithm (PFEGA) [27], and the depth text similarity fusion algorithm (DTSFA) was conducted as a comparative test. In this section, the test set is selected through the public TREC Question Classification (TQC) data set. The experimental results of three different system algorithms are shown in Table 3.

According to Table 3, the algorithms of those different QA systems calculate the information of the matching texts from different aspects. Specifically, compared to the depth evidence score fusion algorithm, the Watson system evidence-scoring algorithm is lacking the pretreatment process for Chinese textual content, so the calculating results of the textual similarity are easily disturbed by the excessive noise. WSESA is used to calculate the textual information from four aspects: word frequency (by PTM), word order (by TA), syntax (by S-B), and structure (by LFACS). Lexical features in PFEGA include *n*-gram, word shape, question length, and so on. The other three feature algorithms in PFEGA are used to calculate from three aspects: syntax (by *syntactic features*), semantics (by *semantic features*), and structure (by *structural features*). Whereas this paper studies the word frequency, word order, TF-IDF, syntax, structure, and semantics of text information in the depth evidence score fusion algorithm. According to the experimental results, the depth evidence score fusion algorithm can better compute the multifeature similarity between the matching texts and give the accurate score of the textual similarity between the matching texts by the depth neural network model, and many feature algorithms have been imported especially at the semantic level of the algorithm. The depth evidence score fusion algorithm has a good guarantee for the accuracy of the similarity calculation in the QA system. Moreover, it can be well applied to the processing of the textual information and the textual classification of each module of the QA system. Through the deep neural network model, the depth evidence score algorithm can be used to the multifeature evidence scoring for the corresponding textual information and give the accurate score of the evidence paragraph. The depth of evidence score fusion algorithm is guaranteed to ensure that the QA system can provide optimal candidate answers.

## 5. Conclusion

In this paper, a depth text similarity fusion algorithm for Chinese medical intelligence QA system is proposed. In this algorithm, the word preprocessing in the text is performed, and the scores of the similarity degree are calculated between the matching texts. The similarity of information between texts is ultimately calculated deeply by the depth neural network model. Considering the complexity of Chinese context and the diversity of Chinese semantics as well as the network demand of social health care, the intelligent QA system needs to output the answers that are required by users quickly and accurately from the massive medical field data. The depth text similarity fusion algorithm is however far beyond one's reach at the semantic level. Whether it is for the domain specific or open domain, there are many problems in the Chinese question answering system. Therefore, how to optimize the Word2vec training model and dig out the similarity between the deep semantic layers of the matching texts will be crucial problems in the future work.

## Figures and Tables

**Table 1:** Depth text similarity fusion algorithm scoring results.

	*S* (*Q*, *P*1)	*S* (*Q*, *P*2)	*S* (*Q*, *P*3)	*S* (*Q*, *P*4)
*p*_1_	0.403	0.575	0.816	**0.911**
*p*_2_	0.366	0.585	0.734	**0.767**
*p*_3_	0.281	0.618	0.789	**0.848**
*p*_4_	0.445	0.355	0.563	**0.619**
*p*_5_	0.793	0.726	0.726	**0.837**
*p*_6_	0.474	**0.145**	**0.785**	**0.769**
*S*	0.428	0.506	0.779	**0.852**

**Table 2:** Node corresponding to join the evidence score algorithm.

*B*	Text word frequency score algorithm
*C*	Text word order score algorithm
*D*	Text TF-IDF score algorithm
*E*	Text syntax score algorithm
*F*	Text structure score algorithm
*G*	Text semantic score algorithm

**Table 3:** Accuracy comparison of different system algorithms.

System	Feature algorithm (%)	Precision (%)	Recall (%)	*F*-measure (%)
WSESA	PTM	73.7	91.8	81.7
	S-B	81.4	90.4	85.7
	TA	75.3	84.9	79.8
	LFACS	86.2	57.5	69.0

PFEGA	Lexical features	57.2	62.4	59.68
	Syntactic features	63.7	79.8	70.84
	Semantic features	71.8	84.6	77.67
	Structural features	68.6	82.2	74.78

DTSFA	Frequency	76.8	92.7	84.0
	Order	74.5	82.3	78.21
	TF-IDF	83.4	75.1	79.03
	Syntax	85.8	88.7	87.23
	Structure	72.3	83.4	77.46
	Semantics	**80.6**	**77.2**	**78.86**

## Data Availability

The data used to support the findings of this study are available from the corresponding author upon request.
